# Ethnobotanical study of medicinal plants utilized in the management of candidiasis in Northern Uganda

**DOI:** 10.1186/s41182-022-00471-y

**Published:** 2022-10-14

**Authors:** Betty Akwongo, Esther Katuura, Anthony M. Nsubuga, Patience Tugume, Morgan Andama, Godwin Anywar, Mary Namaganda, Savina Asimwe, Esezah Kyomugisha Kakudidi

**Affiliations:** 1 Department of Plant Science, Microbiology and Biotechnology, School of Biosciences, College of Natural Sciences, Makerere University, P. O. Box 7062, Kampala, Uganda; 2 Department of Biology, Faculty of Science, Muni University, P.O. Box 725, Arua, Uganda

**Keywords:** *Candida* species, Health risks, Medicinal plants, Oropharyngeal candidiasis, Vulvovaginal candidiasis

## Abstract

**Background:**

The emergence of resistant *Candida* species to antifungal drugs has led to resurgence in herbal usage globally. However, little is known about anti-candida plants. This study explored ethnomedicinal plants as treatment option for candidiasis in Pader, Northern Uganda.

**Methods:**

A cross-sectional survey of potential anti-candida plants was conducted using questionnaires, focus group discussions and field observations in March 2022. Sixty-three respondents were selected by snowball technique. The frequencies of respondents/responses were analyzed, associations of respondents’ socio-demographics with indigenous knowledge of herbal usage established by Chi-square (*χ*^2^) test using SPSS 27. Informant Consensus Factor was computed to establish level of agreement on herbal usage, and thematic analysis done for focus group discussions.

**Results:**

Candidiasis is still common and troublesome in Pader. All herbalist had equal chances of receiving and treating candidiasis patients irrespective of herbalist’s gender, age, education level, occupation, marital status and religion (*p* > 0.05). About 39.7% of herbalists received candidiasis patients weekly (*p* < 0.01). All herbalists had knowledge on candidiasis. Death (56.8%) and discomfort (36.8%) were the major health risks of oropharyngeal candidiasis (OPC) and vulvovaginal candidiasis (VVC), respectively. A total of 32 potential anti-candida plant species in 18 families were identified. Families of Fabaceae (9 species) and Asteraceae (5 species) had most plant species. Trees (50.0%) and herbs (43.8%) were the dominant life forms. The commonest plants by frequency of mention were: *Momordica foetida* (26)*, Sansevieria dawei* (20)*, Khaya anthotheca* (15)*, Piliostigma thonningii* (10), *Clerodendrum umbellatum* (7), *Hallea rubrostipulata* (5) and unidentified plant, ‘Agaba/daa layata’ in Acholi dialect (5). Plant parts mainly used were roots (56.3%) and stem barks (15.6%) harvested majorly by cutting (46.9%) and uprooting (12.5%). Most respondents (females, 95%) preferred herbal to western medication (*p* < 0.01) due to its perceived effectiveness. There was high consensus among herbalists on herbal remedies for OPC and VVC (FIC = 0.9).

**Conclusions:**

Pader communities have diverse indigenous knowledge on candidiasis and prefer herbal medicines to orthodox treatment for candidiasis. However, the herbalists use unsustainable harvesting techniques like uprooting whole plants and cutting main roots. Hence, the need to document such indigenous knowledge before being lost for community usage and scientific validation.

## Introduction

The global incidence of fungal infections averaged one billion people with more than 1.6 million deaths associated with overall fungal infections [1]. Additionally, there were more than 1.5 million deaths from invasive fungal infections yearly [2]. *Candida* species are the most common cause of serious invasive fungal infections, and contribute significantly to global human morbidity and mortality [3]. Research showed that most fungal infections are caused by *Candida, Aspergillus* species and members of the order Mucorales [4]. In Africa, there is still limited data on fungal disease prevalence since the infections have been inextricably tied to and associated with tuberculosis and HIV [5, 6]. In Uganda, the situation is not any different since 2.5 million people (6.5% of the total population) get fungal infections and about 38,000 people die yearly, mainly from HIV-related fungal infections [7]. Most of these cases are from Eastern and Northern Uganda [8]. These two regions also have the highest burden of HIV-related opportunistic infections, especially oral candida [8].

According to Achkar and Fries [9], the most common infectious agent of candida species is *Candida albicans*. This commensal yeast colonizes the skin, mouth, gastrointestinal and the reproductive tracts. However, other *Candida* species such as *C. glabrata*, *C. tropicalis*, *C*. *parapsilosis, C. kruse,* and *C. guilliermondii* are also increasingly becoming more relevant, since they can also colonize human mucocutaneous surfaces [10]. Mucocutaneous candidiasis is divided into two: (i) non-genital disease with oropharyngeal candidiasis as the most common, and (ii) genitourinary disease frequently manifested as vulvovaginal candidiasis (VVC), which is also termed as vaginal yeast infection or vulvovaginitis in women, balanitis and balanoposthitis in men, and candiduria in both sexes [9]. Uganda registers 45,000 cases of HIV-related oral and oesophageal candidiasis annually. Conversely, non-HIV candida in expectant mothers affects 651,600 women yearly, out of which, 375,540 women experience recurrent episodes per year [7].

In Uganda, the main treatment for fungal infections is fluconazole, which is a broad spectrum anti-fungal drug [11]. However, there is a worrying concern of the emergence of resistant candida strains to this first-line drug [12]. Evolution of multidrug-resistant fungal organisms could lead to complicated human fungal diseases [13].

It is worth noting that an estimated 80% of the population in developing countries particularly in Africa use herbal remedies to treat various ailments including fungal infections [14]. Most rural Ugandans use herbal medication to meet their healthcare needs in a culturally appropriate manner [15]. Studies recently conducted in Uganda revealed that many plant species, for example, *Erythrina abyssinica*, *Pentas longiflora, Albizia coriaria*, are used to treat fungal skin infections [16–20].

In Pader district, the management of fungal infections is a big challenge due to inadequate technical and health infrastructural capacities. This problem unfortunately frustrates efforts to access fungal diagnostic and treatment services [21]. Similarly, the long political instability of 1986–2006 that arose from the conflict between the Lord’s Resistance Army (LRA) rebels of Northern Uganda, and the Uganda People’s Defense Forces (UPDF) greatly disrupted economic activities in the district [22]. This contributed to the negative shift in nutritional contents of the foods for the community leading to malnutrition; and coupled with increased stress, negatively alters people’s (fungal hosts) immune responses. These consequently favor candida species to overgrow and cause candidiasis [23]. Additionally, the war also resulted into serious shortages of healthcare workers, drugs and food supplements [22, 24]. Thus, patients often had to walk long distances in search of healthcare services [25]. This inaccessibility to the modern health care system makes people resort to the use of herbal medicine as an alternative treatment option [26]. Traditional Medical Practitioners (TMP) are custodians of a lot of valuable knowledge on medicinal plants for treatment of many diseases including fungal diseases. Unfortunately, some of this knowledge has not been documented.

As a result, there is increasing loss of indigenous knowledge on medicinal plants in Africa in general and Uganda in particular, due to their death [27]. This implies that the undocumented information may be permanently lost [28]. The situation is compounded by the fact that the custodians of Traditional Medicine (TM) are often secretive and accessing such valuable information may not be easy [27]. However, ethnobotanical studies on traditional medicinal plants usage for treatment of various ailments, for example candidiasis, are not comprehensive enough in several African countries such as Uganda where Pader district is located. As a result, there is need for more research in this area in order to fill the gaps [29]. It is hoped that when such studies are made, they will help in documenting and preserving this valuable knowledge for continued use by the community, as well as for future use in research activities such as testing for antifungal compounds. This will lead to attainment of Sustainable Development Goal, agenda 3, of good health and well-being by 2030.

This study therefore documented potential anti-candida plant species, plant parts used, life forms, as well as the indigenous knowledge of potential anti-candida herbal remedies in Pader district.

## Methods

### Study area

**Fig. 1:**
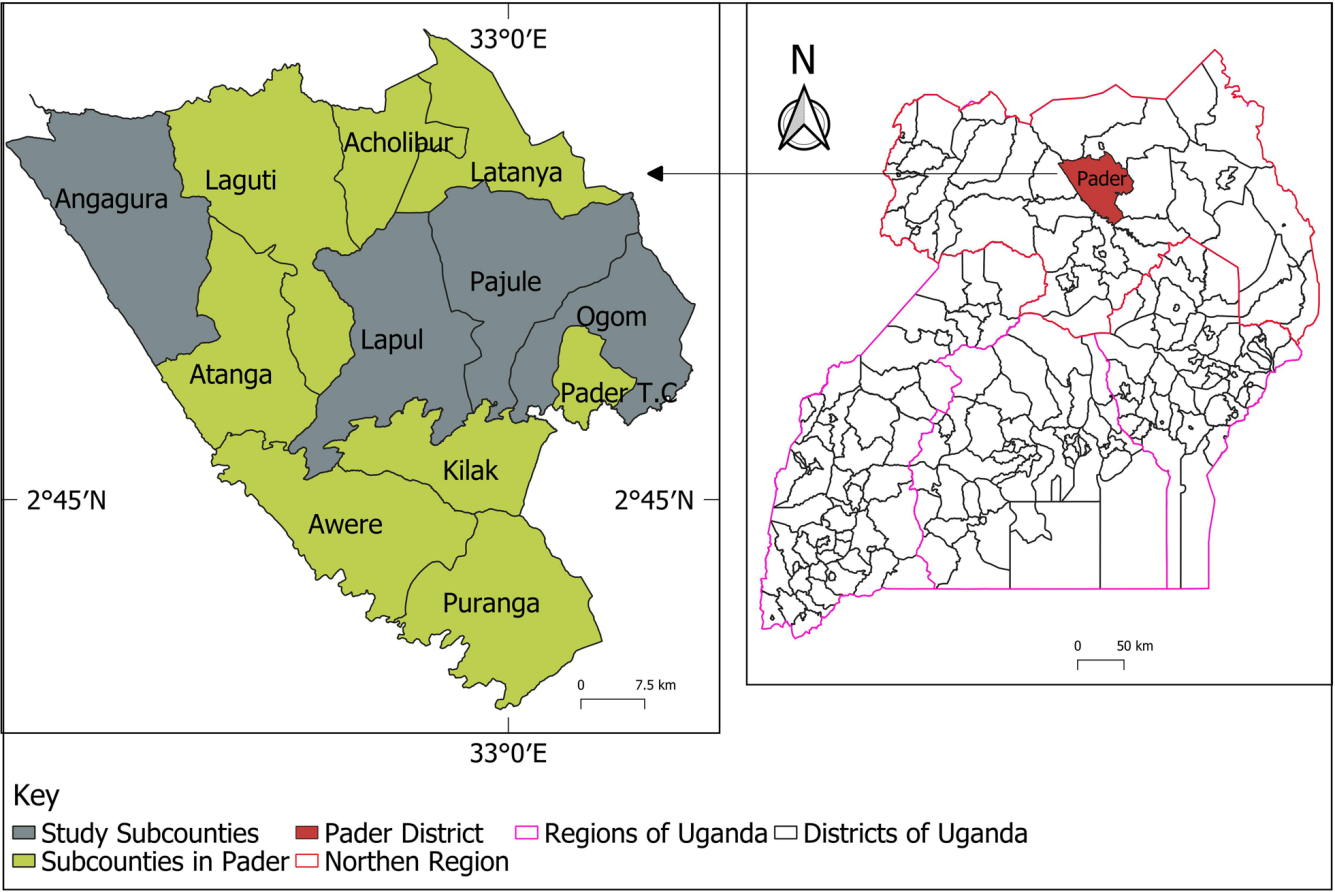
Study sub-counties in Pader district, Northern Uganda (Source: QGIS 3.2 Bonn)

The research was conducted in Pader district in Northern Uganda in March 2022. The district lies between 32°45′E–33°00′E and 2°45′N–3°00′N. Pader district is characterized by gender inequality, high youth unemployment, low economic development and inaccessibility to basic services including education and health [30]. Overall, 31.6% of the households do not have access to health facilities [31]. Out of the 12 sub-counties (Angagura, Laguti, Acholibur, Latanya, Atanga, Lapul, Pajule, Ogom, Pader, Pader Trading Centre, Awere and Puranga) in Pader district, Ogom, Angagura, Lapul and Pajule were chosen for this study (Fig. 1). This is because Ogom and Angagura Sub-counties have the poorest access to health facilities at 71.7% and 58.4%, respectively. These families are located more than 5 km away from the nearest health centers [31]. On the other hand, many communities in Pajule and Lapul sub-counties, can easily access Pajule Health Centre (HC IV) which is the biggest community hospital in Pader district. It is also important to note that Ogom and Angangura sub-counties have the highest and third highest number of food insecure households at 27.4% and 25.3% in the district [31]. The poor living conditions experienced by such communities predispose them to opportunistic diseases including fungal infections.

### Research design

The study was a cross-sectional survey that involved collection of ethnobotanical information on utilization of medicinal plant species in the management of candidiasis in Pader district. Both qualitative and quantitative approaches (mixed methods) were used. Focus group discussions (FGD) and semi-structured interviews (survey and open-ended questions) were used to collect in-depth information on anticandidal medicinal plant usage.

### Data collection methods

#### Sampling strategies

The principle of saturation was followed during snowball sampling to select 63 key respondents (herbalists and knowledgeable persons) for key informant interviews. Selection of respondents was based on the recommendations of local authorities/ community elders. Herbalists had to have at least 5 years experience [32] of treating candidiasis using potential anti-candida herbal remedies for them to be included in the study. Eight to ten pregnant women in antenatal visits at each of the health centers in the study sub-counties were randomly chosen for FGD using interview guides [33].

#### Field survey

Semi-structured interviews using questionnaire guides were carried out among the herbalists. The questionnaire covered a list of topics on potential anti-candida plant species. The initial respondent was selected randomly by flipping a coin [34]. Snowball sampling was used to identify the 63 subsequent herbalists in their networks [35]. Rapport was created with the herbalists by researchers through establishment of cordial interpersonal interactions on the intentions of the study [36]. Field excursions were undertaken with the help of the herbalists to locate the medicinal plants in their habitats. Questionnaire guides were administered via personal contact discussions [37]. In order to obtain all the relevant data on herbal remedies against VVC and OPC, data were collected until saturation of views of respondents was reached [38]. With permission from the respondents, the interviews were audiotaped in order to obtain accurate information [39]. Field guides familiar with the areas were used to identify the selected respondents. Data collection was done with the help of field assistants who were knowledgeable in the local language. Data for FGD were collected using interview guides. The researchers facilitated the discussions among the participants [40]. All the plants mentioned were collected following standard procedures described in Martin [34], voucher specimens were prepared and taken for identification and classification at the Makerere University Herbarium. Current taxonomic nomenclature was used based on the African Plant Database (APDB), Global Biodiversity Information Facility (GBIF) and Plants of the World (POWO). The voucher specimens were given voucher numbers and deposited at Makerere University Herbarium.

### Data analysis

Data were analyzed using SPSS 27. Descriptive statistics such as frequencies were used to present respondents’ demographics, knowledge of candidiasis and potential anti-candida herbal remedies in tables, bar graphs and pie charts. Associations of respondents’ socio-demographics with knowledge of herbal usage were established using Chi-square (*χ*^2^) test at 5% level of significance. These statistics were used to identify the most useful plant species for treating OPC and VVC [41].

Informant Consensus Factor (FIC) was computed to establish the agreement among herbalists on potential anti-candida herbal usage. FIC values range from 0 to 1, with 1 indicating highest level of informant consensus/agreement and 0 for no agreement on use of medicinal plant species for particular ailments. FIC was calculated according to formula, FIC = Nur − Nt/Nur − 1 [42]

Where, Nur = number of use reports from informants for a particular plant use category, Nt = number of species that are used for that use category for all informants. Values of FIC above 0.7 were used to indicate high levels of agreement of traditional knowledge usage of medicinal plants [43].

FIC analysis was done by grouping medicinal plant species into three categories, that is, plants for treating OPC only, VVC only and both OPC and VVC conditions. The FGD responses were transcribed and thematic analysis as used by Omara and Akwongo [44] was used to generate themes and sub-themes from the discussions. Some of the key findings (verbatim) with illustrative experiences were incorporated into the discussion section [45] to give insights of candidiasis in the study area.

## Results

### Socio-demographic characteristics of respondents (herbalists)

The majority of the respondents were female (95.2%), in the age bracket of 36–55 years (39.7%), attained primary education (66.7%) and were crop farmers (88.9%). Furthermore, most respondents were Roman Catholics (68.3%) and married (85.7%) (Table 1).

**Table 1:** Socio-demographic characteristics of herbalists

Demographics	Frequency (*N* = 63)	Percentage
Gender		
Female	60	95.2
Male	3	4.8
Age (years)		
18–35	18	28.6
36–55	25	39.7
> 55 years	20	31.7
Education level		
None	15	23.8
Primary	42	66.7
Secondary	6	9.5
Main occupation		
Crop farming	56	88.9
Others	7	11.1
Religious affiliation		
Roman catholic	43	68.3
Anglican	9	14.3
Pentecostals	11	17.5
Marital status		
Single	1	1.6
Married	54	85.7
Divorced	1	1.6
Widowed	7	11.1

### Herbalists’ knowledge on candidiasis (signs and symptoms, and health risks) and herbal usage

**Fig. 2:**
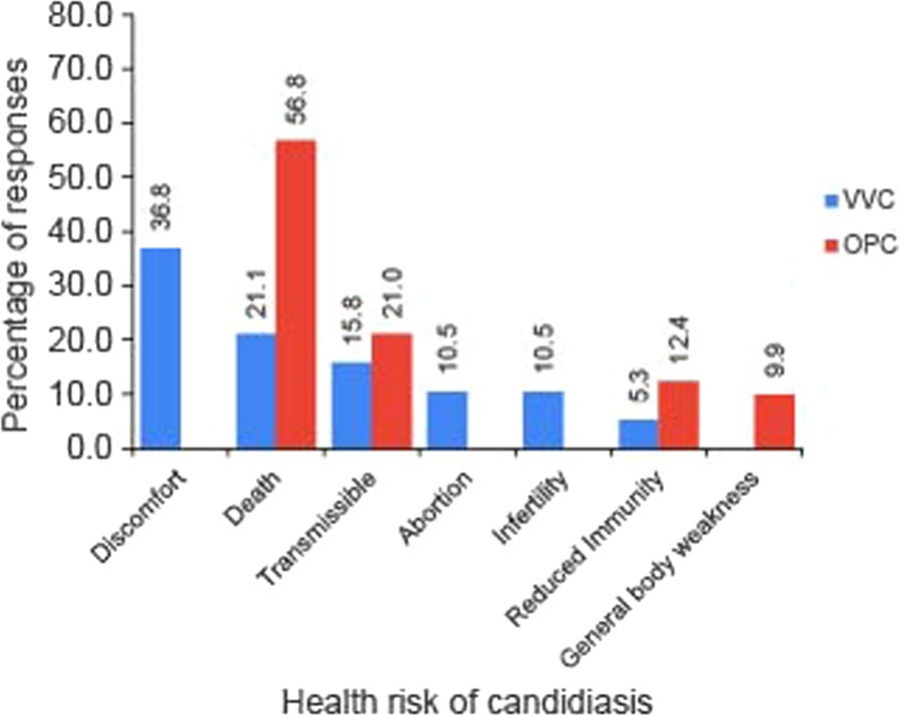
Health risks of candidiasis

**Fig. 3:**
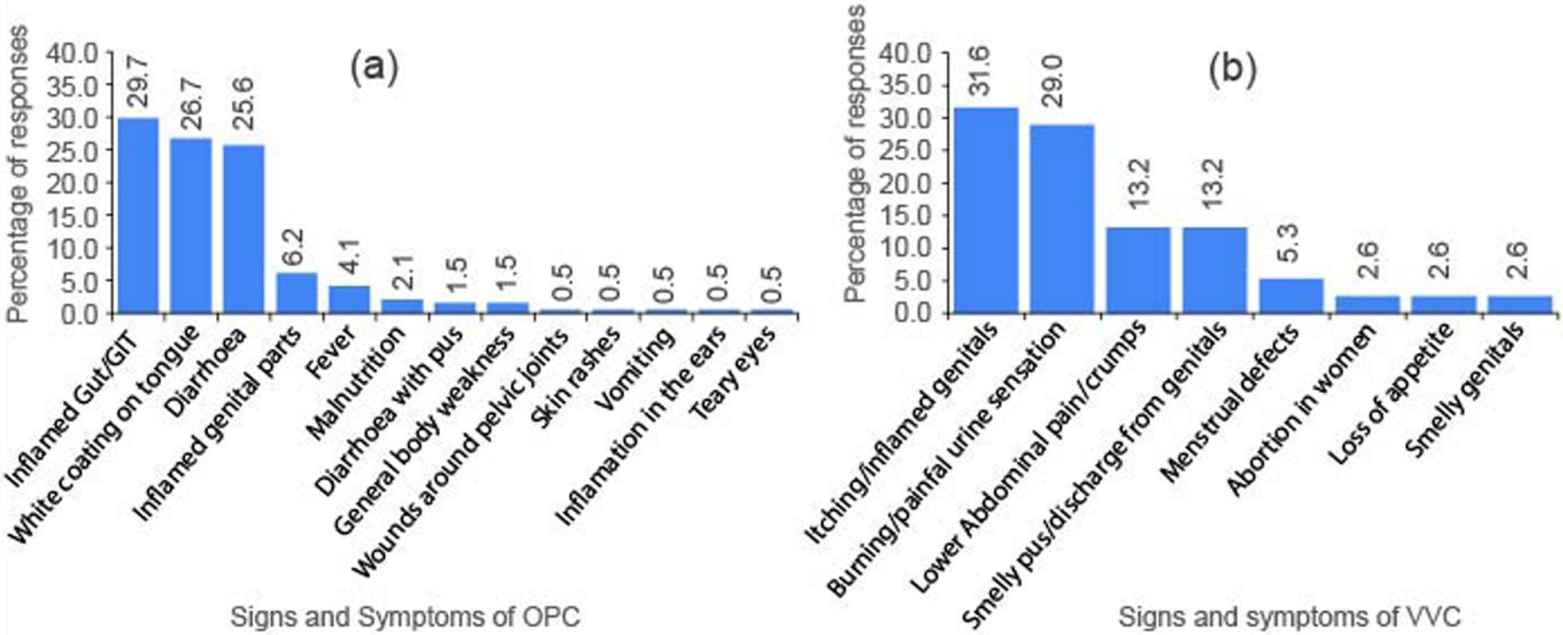
Signs and symptoms of oropharyngeal candidiasis (**a**) and vulvovaginal candidiasis (**b**)

Based on herbalists’ experiences from interactions with the community, and also from the direct/indirect exposure to the disease(s); all respondents had knowledge on candidiasis. Death (56.8%) and discomfort (36.8%) were reported as the main health risks for OPC and VVC, respectively (Fig. 2). The main reported signs and symptoms of OPC (Fig. 3a) were inflammation of the gut (29.7%), white coatings on the tongue (26.7%) and diarrhea (25.6%); while for VVC, the signs and symptoms indicated were; itching genitals that later become inflamed due to scratching (31.6%), burning/ painful sensation when urinating (29.0%); lower abdominal pain/ cramps (13.2%) and smelly discharge from the genitals (13.2%) (Fig. 3b).

**Fig. 4:**
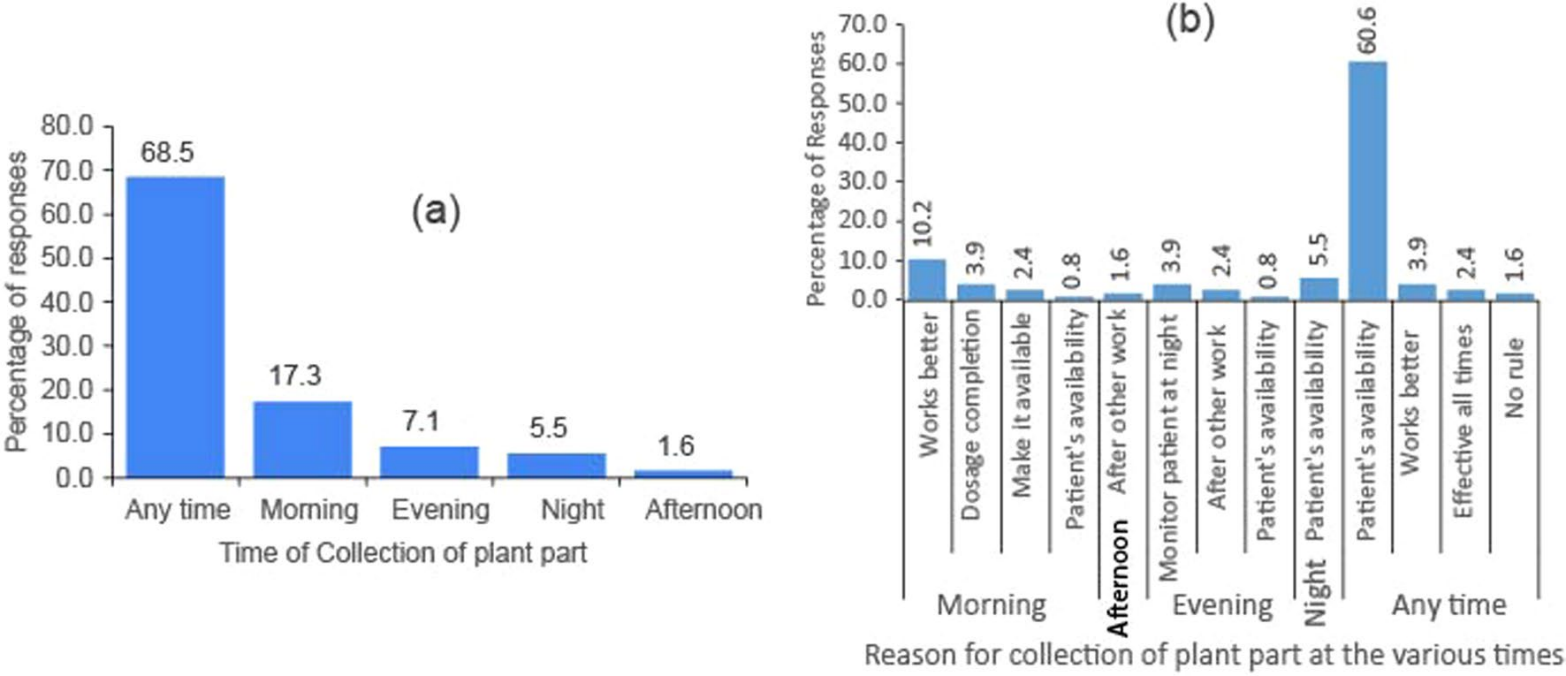
Plant collection time (**a**) and reasons (**b**)

Most herbalists collected the potential anti-candida plants at anytime (68.5% (Fig. 4a) citing patients’ availability (60.6% (Fig. 4b) as the main reason. This was followed by collecting the plants in the morning hours (17.3%), with the main reason being that the plants work better when picked at that time (10.2%).

### Burden of candidiasis in the study area

There was no association between herbal treatment frequencies of candidiasis with each of the demographic characteristics of the herbalists (*p* > 0.05). However, most herbalists (*p* < 0.01) received candidiasis patients weekly (25), followed by monthly (19) (Table 2).

**Table 2:** Frequency of herbal treatment of candidiasis in relation to herbalists’ socio-demographics

Characteristics	Categories	Daily	Once a week	Twice a week	Thrice a week	After 2 weeks	Monthly	Chi-square (*χ*^2^)	df	*p*-value
Gender	Female	7	24	7	2	2	18	1.649	5	0.895
	Male	1	1	0	0	0	1			
Age (years)	18–35	1	8	3	1	0	5	6.852	10	0.739
	36–55	2	10	3	1	1	8			
	> 55	5	7	1	0	1	6			
Education level	None	2	4	2	0	0	7	7.292	10	0.698
	Primary	6	17	5	2	2	10			
	Secondary	0	4	0	0	0	2			
Main occupation	Crop farming	7	21	6	2	2	18	1.85	5	0.869
	Others	1	4	1	0	0	1			
Marital status	Single	0	1	0	0	0	0	10.208	15	0.806
	Divorced	0	0	0	0	0	1			
	Widowed	2	3	0	0	1	1			
	Married	6	21	7	2	1	17			
Religious affiliation	Roman catholic	8	16	3	0	2	14	11.628	10	0.307
	Anglican	0	4	2	1	0	2			
	Pentecostal	0	5	2	1	0	3			
Overall frequency of treatment of candidiasis		8	25	7	2	2	19	42.429	5	0.000

### Treatment options for candidiasis

Treatment options for candidiasis was highly influenced by gender (*p* < 0.01) but not age, educational level, occupation, marital status or religious affiliation (*p* > 0.05). More females (59) preferred herbal medicine to western medication (*p* < 0.01) than males (2) (Table 3).

**Table 3:** Treatment options for candidiasis in relation with gender, age, educational status, occupation, marital status and religion

Characteristics	Categories	Treatment options for candidiasis			Chi-square (*χ*^2^)	df	*p*-value
		Western medication	Herbal remedies	Western and herbal			
Gender	Female	0	59	1	20.346	2	0.000
	Male	1	2	0			
Age (years)	18–35	0	18	0	3.697	4	0.449
	36–55	0	24	1			
	> 55	1	19	0			
Education level	None	0	15	0	1.033	4	0.905
	Primary	1	40	1			
	Secondary	0	6	0			
Main occupation	Crop farming	1	54	1	0.258	2	0.879
	Others	0	7	0			
Marital status	Single	0	1	0	0.344	6	0.999
	Divorced	0	1	0			
	Widowed	0	7	0			
	Married	1	52	1			
Religious affiliation	Roman catholic	1	42	0	5.245	4	0.263
	Anglican	0	9	0			
	Pentecostals	0	10	1			
Overall treatment options for candidiasis		1	61	1	114.286	2	0.000

### Herbalists’ knowledge of herbal medicine

The source of knowledge on potential anti-candida plants is not associated with gender, age, education level, main occupation, marital status and religious affiliations (*p* > 0.05). However, the majority of the respondents (*p* < 0.001) inherited the knowledge from relatives (35), followed by fellow herbalists (19) (Table 4).

**Table 4:** Target group versus source of information on herbal medicine

Target group (herbalists)		Source of knowledge on herbal medicine				Chi-square (*χ*^2^)	df	*p*-value
Characteristics (*n* = 63)	Categories	Inherited (relatives, e.g., parents/grandparents, uncles)	Fellow herbalists	Dream	Self-discovery			
Gender	Female	33	18	7	2	0.531	3	0.912
	Male	2	1	0	0			
Age (years)	18–35	10	6	2	0	5.255	6	0.512
	36–55	15	8	2	0			
	> 55	10	5	3	2			
Education level	None	9	4	1	1	7.127	6	0.309
	Primary	20	15	6	1			
	Secondary	6	0	0	0			
Main occupation	Crop farming	30	19	6	1	5.866	3	0.118
	Others	5	0	1	1			
Marital status	Single	1	0	0	0	5.287	9	0.809
	Divorced	1	0	0	0			
	Widowed	2	3	2	0			
	Married	30	16	5	2			
Religious affiliation	Roman catholic	25	11	5	2	2.283	6	0.892
	Anglican	4	4	1	0			
	Pentecostals	6	4	1	0			
Overall source of knowledge		35	19	7	2	41.063	3	0.000

### Medicinal plants usage by herbalists for management of candidiasis in Pader district

#### Reasons for choice of herbal remedies

**Fig. 5:**
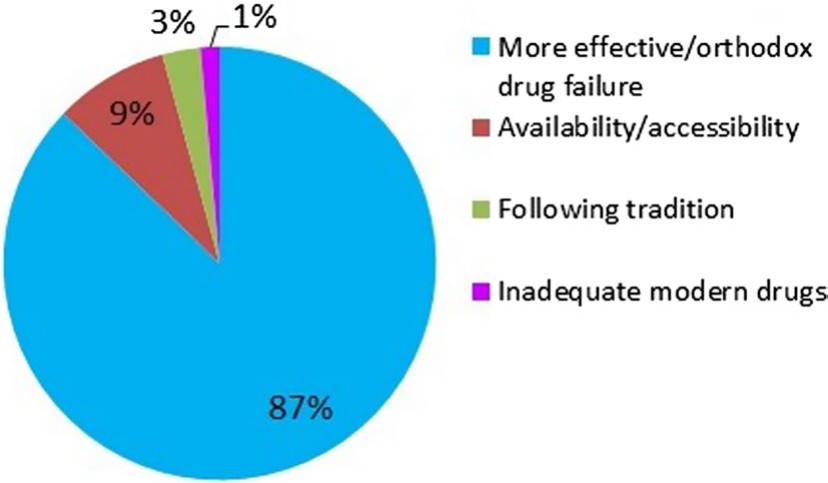
Reasons for choice of herbal remedies

The majority of the respondents preferred herbal medication to conventional medicine mainly due to its effectiveness/ failure of orthodox drugs from hospitals (87%) (Fig. 5 and Table 7).

#### Informant consensus factor for candidiasis treated by herbalists

FIC values for medicinal plants for treatment of only OPC or VVC were below 0.7, indicating low agreement among the herbalists on potential anti-candida plants for management of either of the infections, while FIC for medicinal plants used for treatment of both OPC and VVC, was greater than 0.7 (0.9), thus very strong agreement among the herbalists on usage of similar plants to manage both common forms of candidiasis (Table 5).

**Table 5:** Informant consensus factor (FIC) for usage of potential anti-candida plants

Most common candidiasis	Number of plant species (Nt)	Number of citations (Nur)	Fic
OPC	19	42	0.56
VVC	4	6	0.40
OPC/VVC	9	79	0.90

#### Medicinal plants for treatment of candidiasis

A total of 32 potential anti-candida plant species belonging to 18 families were identified. The majority of the plant species belonged to family Fabaceae (9 species,  50.0%) and Asteraceae (5 species, 27.8%). Half of all the plant species (16, 50.0%) were trees, followed by herbs (14 species, 43.8%). Roots (56.3%) were the most commonly used plant part followed by stem barks (15.6%) and lastly leaves (3.1%). However for a similar plant, more than one plant part was also used i.e., barks/roots (9.4%), leaves/roots (9.4%), fruits/roots (3.1%) and stems/leaves (3.1%). The most commonly used plant species were *Momordica foetida* (26), *Sansevieria dawei* (20), *Khaya anthotheca* (15), *Piliostigma thonningii* (10), *Clerodendrum umbellatum* (7), *Hallea rubrostipulata* (5) and unidentified plant, ‘Agaba/daa layata’ in Acholi (5). Half of all the respondents, most of whom were herbalists (21), harvested the medicinal plants by cutting (46.9%) followed by uprooting (12.5%) and digging (12.5%). All the medicinal plants species were obtained from the wild, except *S. dawei* which was not only wild but also cultivated. Herbs were reported to be abundant during rainy season, while trees were available throughout the year (Table 6).

**Table 6:** Medicinal plants usage for management of candidiasis in Pader district

Family and scientific names/voucher number	Local names	Hb	Part/s used	Life form	Season when abundant	Harvesting method	Freq	Disease treated	Mode of preparation (Prep.) and administration (Admn.)
ANACARDIACEAE Sclerocarya birrea (A. Rich.) Hochst BA028	Otitimo^A^	gd	Bark	Tree	Throughout the year	Cutting	1	OPC/VVC	Prep: maceration: wash, remove outer epidermal layer, pound, add little water, sieve Admn: orally: children; 3 tsp3 times a day. Adults; any dose
APOCYNACEAE *Carissa spinarum* L BA027	Acuga^A^	ah	Root/fruit	Shrub	Throughout the year	Cutting/ hand picking	1	OPC	Prep: decoction: wash, pound, boil, cool; fruits: cook, sieve, filtrate cooked with porridge Admn: orally: children: 2 tsp thrice daily. Porridge to be drunk as much as desired. Anally: 4 full syringes (10 ml auto disable) twice a day & the child immediately passes green stool, medication continues green diarrhoea stops
ASTERACEAE									
*Baccharoides adoensis* (Sch. Bip.) H. Rob BA007	Ludia^L^	bh	Root/ leaves	Herb	Rainy season	Uproot / cut leaves	2	OPV/ VVC	Prep: maceration/decoction: wash; for roots, peel the bark, pound, mix with moderately cooled boiled water. For leaves, boil with moderate water covering it, sieve, add little Sugar Admn: orally: children < 2 years; ½ tsp 3 times daily; 2–5 years; 1 tsp 2 or 3 times daily. Adults; 2 tsp twice a day for about 2 weeks. Anally: children: < 2 years; 2-3 mls 3 times daily; 2–5 years; 5mls 3 times daily
*Bidens pilosa* L BA031	Labika^A^	gd	Leaves	Herb	Rainy season	Hand picking	1	OPC	Prep: decoction: wash, boil until ready, cool, then sieve Admn: orally: 2 tsp3 times a day
*Cyanthillium cinereum* (L.) H. Rob BA008	Lacaka^L^	gd	Stem/ leaves	Herb	Rainy season	Uprooting	1	VVC	Prep: maceration: wash, pound, mix with water and sieve Admn: bath/smear: pour filtrate in bathing water/ smear filtrate around vulva after bathing, 3 times a day
*Echinops sp.* BA024	Atyiita^A^	gd/Bh	Leaves/ root	Herb	Rainy season	Uprooting / Hand picking	3	VVC	Prep: maceration: wash, can pound and mix with little water Admn: orally: children; squeeze out few drops in the mouth, 3 times a day Adults: chew few/ drink any amount of filtrate once/ thrice daily. Bath: add filtrate in bathing water
*Solanecio mannii* (Hook. F.) C. Jeffrey. BA003	Taa lyec^A^	bh	Root	Herb	rainy season	dig out root	2	OPC/VVC	Prep: maceration: wash, pound, add little water & squeeze to get filtrate Admn: orally: orally: children 3 tsp twice a day; adults, ¼ mug cup once a day
FABACEAE *Piliostigma thonningii* (Schumach.) Milne-Redh. BA009	Ogali^A^	Bh	Root/ bark	Tree	Throughout the year	Cutting	10	OPC	Prep: maceration/decoction: can remove the epidermal layer, pound, mix with little water/ boil & cool, sieve Admn: orally: 1 or 2 tsp/ 5/10/15 mls once/twice/thrice a day. Can mix little filtrate with food; anally: 3mls / 1 full syringe (10 ml auto disable) once a day
CELASTRACEAE *Mystroxylon aethiopicum* (Thunb.) Loes. BA017	Akekedo^A^	hc	Root	Tree	Throughout the year	Cutting	1	VVC	Prep: maceration: pound, mix with moderate water, sieve Admn: orally: adult; 1 nice cup once a day. Youths, ½ nice cup once a day
COMBRETACEAE *Combretum molle* R.Br. ex G.Don. BA022	Olim^A^	bh	Bark	Tree	Throughout the year	Cutting	1	OPC	Prep: maceration: pound, mix with water, sieve Admn: orally: 2 tsp twice daily
CONVOLVULACEAE									
*Astripomoea malvacea* (Klotzsch) A. Meeuse. BA006	Agila^L^ / Temony^A^	bush	Root	Herb	Rainy season	Uprooting	3	OPC	Prep: maceration/decoction: wash, can remove epidermal layer, peel bark, pound them, mix with little cold water/ boil and cool, sieve Admn: orally: children; ½ or 1 tsp twice/ thrice a day, for one week. Adults; any dose any time. Anally: 1 full syringe (10 ml auto disable) twice a day
Unidentified BA029	Agaba/daa layata^A^	bh/ gd	Root	creeping herb	rainy season	uprooting/ digging out/ cutting	5	OPC/VVC	Prep: maceration: wash, remove epidermal skin by peeling, pound, mix with little/moderate water, sieve Admn: orally: children: 1 or 2 or 3 tsp twice/thrice a day Adults: 2 tsp/ 50 mls / ¼ cup once/twice a day for 1 week/any dose; Massage affected area Brushing off fungal coating with residue
CUCURBITACEAE *Momordica foetida* Schumach BA005	Bomo^A^	bh	Root	Climbing herb	Rainy season	Uprooting/ cutting roots	26	OPC/VVC	Prep: maceration/decoction: wash, can remove outer epidermal layer, pound, mix with cold water or boil, cool then sieve Admn: orally: children; 1 or 2 or 3 tsp 2 or 3 times a day. Adults; engulf filtrate full in the mouth and swallow it, twice a day. Anally: children 3 or 5 or 10 mls or 1 or 2 or 3 full syringes (10 ml auto disable) twice/ thrice a day for a week/10 days/until recovery. Massage: massage stomach with residue. Bath: add filtrate in bathing water
DRACAENACEAE *Sansevieria dawei* Stapf BA002	Twooro bye / gwok^A^	ah	Root	Herb	Throughout the year	Uprooting/ cutting or digging out (roots unsustainably removed that cause the plant to dry out	20	OPC/VVC	Prep: maceration/ decoction: Wash, can remove epidermal layer, pound mix with little cold water/boil until turns yellow, cool, sieve (can also be mixed with *Momordica foetida*, *Hallea rubrostipulata)* Admn: orally: children; 1 or 2 tsp 1 or 2 or 3 or 4 times a day for 2 weeks/until symptoms disappear. Adults; 2 tsp twice a day/any amount, thrice a day. Anally: 5/10/30 mls / 2 full syringes (10 ml auto disable) once / twice/thrice a day. Massage: residue massaged on the stomach Smear: filtrate smeared around reddened anal area/ Pour some filtrate in bathing water Ear drop: add 2 or 3 drops in the ears once a day
EUPHORBIACEAE									
*Acalypha crenata* Hochst. ex A. Rich. BA001	Ayila^A^	cg	Root	Herb	Rainy season	Uprooting (the whole plant removed)	3	OPC/VVC	Prep: maceration/ decoction: cut, wash, remove epidermal skin, pound fresh/dried, power, mix with little cool boiled water/ boil, cool, & sieve Admn: oral route: adults; 1 or 4 nice cup once a day; Children; 1 or 2 tsp twice a day
*Croton macrostachyus* Hochst. ex Delile BA032	Lagwok^A^	bh	Root	Tree	Throughout the year	Cutting	1	OPC	Prep: decoction: wash the root, pound, boil, cool Admn: anally: 3 full syringes (10 ml auto disable) times a day. Complete one day treatment done on weekly basis until recovery
FABACEAE									
*Albizia malacophylla* var. ugandensis Baker f. BA011	Ayeyek^A^	bh	Root	Tree	Throughout the year	Cutting	1	OPC	Prep: maceration: remove epidermal layer, wash, pound, mix with cold water, sieve Admn: orally: two tsp thrice a day for one week. Massage the head with residue
*Erythrina abyssinica* Lam. ex DC BA020	Kicoro/ Lacoro^A^	gd	Bark	Tree	Throughout the year	Cutting	2	OPC	Prep: maceration: remove the epidermal layer, wash, pound, mix with little water Admn: orally: children; 1tsp twice a day. Adult; 2 tsp twice a day for about one week Bath: pour filtrate in bathing water. Can also put few drops in the mouth 3 times a day
*Indigofera arrecta* Hochst. ex A. Rich BA021	Laywe madongo^A^	gd/ bh	Root	Herb	Rainy season	Digging out / cutting	3	OPC	Prep: maceration/decoction: remove the epidermal layer, wash, pound, mix with little water /boil and cool, sieve Admn: orally: 1 tsp twice/ thrice a day; Anally: one full syringe (10 ml auto disable) twice a day
*Indigofera spicata* Forssk. BA012	Lakemtu^A^	afp	Root	Herb	Rainy season	Uprooting	3	OPC	Prep: maceration: wash, remove epidermal layer, pound the bark, mix with moderate cooled boiled water Admn: orally: children: 1 tsp once/twice a day. Adults: 4 tsp twice a day Massage all over the body with more emphasis on the mouth and anal areas)
*Philenoptera laxiflora (*Guill. &Perr.) Roberty. BA018	Olwedo^A^	hc	Root	Tree	Throughout the year	Cutting	1	VVC	Prep: maceration: wash, pound, mix with water, sieve Admn: orally: adults and youths: 1 mini mug once a day
*Senna siamea* (Lam.) H.S.Irwin & Barneby. BA015	Gasia^A^	hc	Root	Tree	Throughout the year	Cutting	1	OPC	Prep: maceration: remove epidermal layer of bark, pound, and mix with little water Admn: orally: 2 tsp twice a day for about 4 days or a week
Unidentified BA023	Amumuru^A^	gd	Root	Tree	Throughout the year	Dig out	2	OPC	Prep: maceration: wash, pound, mix with little water, sieve, can also mix with *Piliostigma thonningii* Admn: orally: 1 tsp thrice a day; Anally: 1 full syringe (10 ml auto disable) twice daily
MELIACEAE *Khaya anthotheca* (Welw.) C.DC BA013	Tido^A^	ft/rp/ gd/as	Bark/root	Tree	Throughout the year	Cutting	15	OPC/VVC	Prep: maceration/decoction/ infusion: remove epidermal layer, pound, mix with little cold/ warm water/ boil & cool, sieve Admn: orally: children: 1 or 2 tsp/3mls for 2 or 3 times a day for about 3 days/ until recovery. Adults: any dose; anally: 1 or 3 full syringe(s) (10 ml auto disable) / 3mls for 2 or 3 times a day. Can also add a drop in the mouth, 3 times a day
FABACEAE *Acacia persiciflora* Pax BA014	Itooko/ladiku^A^	hc	Bark	Tree	Throughout the year	Cutting	1	OPC	Prep: maceration: remove epidermal layer, pound and mix with moderate water Admn: orally: 2 tsp twice a day for 1 week
MORACEAE *Ficus glumosa* Delile BA016	Kworo^A^	hc	Root	Tree	Throughout the year	Cutting	1	OPC	Prep: maceration: pound, mix with very cold water Admn: orally: children; 2 full bottle tops of filtrate, twice a day. Adults; 4 bottle tops of filtrate twice a day
RHAMNACEAE *Ziziphus abyssinica* Hochst. exA.Rich BA010	Okutu lango^A^	gd	Root	Tree	Throughout the year	Cutting	1	OPC	Prep: decoction: remove epidermal layer, wash, pound, boil in water until soft, cool and sieve. Residue mix with boiled cooking oil Admn: orally: children: 1 tsp of filtrate 3 times a day; Massage the stomach with prepared residue
RUBIACEAE *Hallea rubrostipulata* (K. Schum.) Leroy. BA019	Oculup^A^	gd	Root/bark	Tree	Throughout the year	Cutting	5	OPC/VVC	Prep: maceration/decoction: remove epidermal layer, wash, pound, mix with little water/ boil, cool, sieve, can mix with S*ansevieria dawei* Admn: orally: 3mls / any amount 2 or 3 times a day; Anally: 3 mls three times a day
SAPOTACEAE *Vitellaria paradoxa* C.F. Gaertn. BA026	Yaa (Shea butter tree^A^	gd	Bark	Tree	Throughout the year	Cutting	1	OPC	Prep: maceration: remove epidermal layer, pound, add little water, sieve Admn: orally: 1 tsp of filtrate twice daily. Can also add filtrate in food
SIMAROUBACEAE *Harrisonia abyssinica* Oliv. BA025	Pedo^A^	ah	Root	Shrub	Throughout the year	Dig out	1	OPC	Prep: maceration:remove epidermal layer from the root, wash, pound, add little water, sieve Admn: orally: 1 teaspoon twice a day, can also add filtrate in food
LAMIACEAE *Clerodendrum umbellatum* Poir. BA004	Acilo^L^ / Lacer^A^	gd	Leaves/ root	Herb	Throughout the year	Uprooting/ hand picking	7	OPC	Prep: maceration/decoction: wash, can remove epidermal layer, pound, mix with moderate cooled boiled water/ boil until green& cool Admn: orally: Children; 5 mls a day or 1 or 2 tsp 2 or 3 times a day; Adults; 2 tsp 2 or 3 times a day
VITACEAE *Cyphostemma adenocaule* (Steud. ex A. Rich.) Desc. ex Wild & R.B. Drumm. BA030	Anunu^A^	gd	Root	Herb	Rainy season	Digging out	1	OPC	Prep: maceration: wash, pound, add little water, sieve Admn: orally: 1 tsp 3 times a day

Key: local names: Acholi^A^, Langi^L^

Habitats (Hb): Bush: bh; garden: gd; crop garden: cg; anthill: ah; forest: ft; home compound: cp; rocky places: rp; along stream: as; along foot path: afp

Mode of administration: Tea spoon: tsp; OPC; Oropharyngeal candidiasis, VVC; Vulvovaginal candidiasis; Freq. Frequency of mention

#### Modes of preparation and administration

The most commonly used methods of preparation were maceration (56.3%) and decoctions (12.5%). However for a similar plant, more than one method was also used to prepare the herbal remedies i.e., maceration/ decoction (28.1%) or maceration/ decoction/ infusion (3.1%). Water was the only solvent used. In few cases, the tastes of the concoctions were made more palatable by adding sugar. The herbalists used varying measurements of plant materials and water for preparing their medicines. The means of administration included, oral (43.8%) and anal/ rectal routes (for bitter herbs) (3.1%) using disposable syringes.  However more than one mode of administration for a similar plant were also used which included; oral/anal (25.0%), oral/massage (9.4%), oral/bath (6.3%), bath/smear (3.1%), oral/massage/tongue brush (3.1%), oral/anal/massage/bath (3.1%) and oral/anal/massage/ear drop (3.1%). Many respondents gave different and varying doses to children and adults (Table 6).

### Community (non-herbalists) knowledge on candidiasis and their treatment options

The communities in Pader district have great knowledge on candidiasis, which they use to diagnose and offer anti-candida treatment options. They generally prefer herbal to orthodox medication, which herbal treatment they said was cheap and effective (Table 7).

**Table 7:** Focus group discussion to ascertain community (non-herbalists) knowledge on candidiasis and treatment options

SN	Themes	Sub-themes
1	Burden of candidiasis	Every home experienced candidiasis Sometimes recurrent
2	Signs and symptoms of candidiasis	OPC: slippery diarrhoea, pus in stool; reddened anal and genital areas, white coating on tongue, rough feeling of stomach VVC: itching genitals that leads to inflammation, white discharge, painful urination, pus in urine
3	Health risks of candidiasis	OPC: transmissible, death, recurrent VVC: discomfort due to itching; Can become chronic due to poor treatment
4	a) Candidiasis treatment options (orthodox vs herbal medication)	Its trial and error Community in deep villages: heavily use herbal medication Community near health centers (urban and semi urban): use both orthodox and herbal medicine
	b) Preferred anti-candida treatment option	Herbal Reasons: cheap, effective, orthodox drug failure, Side effects of orthodox medication
5	Potential anti-candida plants	*Khaya anthotheca*, *Sansevieria dawei*, *Momordica foetida*, *Clerodendrum umbellatum*, *Hallea rubrostipulata*, *Harrisonia abyssinica* However, VVC is mostly treated using orthodox medication
6	Anti-candida knowledge source	Inherited, elders, herbalists
7	Herbal toxicity	Vomiting; inflammation of the month

## Discussion

### Knowledge of herbalists on candidiasis to justify prospective of potential anti-candida plants

All the herbalists had knowledge on candidiasis. In Acholi dialect, the herbalists referred to OPC as “*two lango*”, while VVC was referred to as “*Odododo*”. *VVC can make you ashamed in public due to itching” (FGD, Ogom HCII).* Nyirjesy et al. [46] reported pain resulting in discomfort as one of the signs of recurrent VVC that creates a very poor self-image that affect the patients psychologically. According to Benzie and Wachtel-Galor [47], herbalists use their knowledge of the diseases to help them diagnose and offer treatment to the patients based on their holistic views and symptoms presented.

### Burden of candidiasis and urgency to use medicinal plants for treatment

Candidiasis disease is still prevalent in Pader district and is one of the causes of death. This finding is in agreement with findings of the research carried out by Ministry of Health (MOH) Uganda and WHO [48] in Pader district, which indicated that OPC was the second leading cause of death among children under five years in internally displaced persons’ camps in Pader district, next to malaria. Furthermore, data from Pader district local government (2022) records (unpublished work), showed an annual general increase in other oral conditions, including OPC in the last 5 years, i.e., 2017–2021. For instance, the year 2020–2021 registered an increase in other oral conditions from 1930 to 2913 cases. The disease burden in the area could be attributed to anti-candida drug resistance, few available anti-candida drugs on the market, and their misuse [49]. Besides, high poverty levels and poor sanitation due to the impact of the 1980s–2008 war [50] predispose the community to opportunistic infections like candidiasis. Thematic analysis of FGD also showed that OPC is still a problem in Pader as reflected in the following quotes: “….*OPC is still very rampant” (FGD, Angagura HCII)*. *It’s now 4 years, the disease has been recurring on my child and not responding to orthodox medication, I don’t know what to do” (FGD, Pajule HCIV,).*

The community generally prefers herbal to orthodox candidiasis medication because the herbs were cheap and effective. The effectiveness of the potential anti-candida plants could be due to the diversity of bioactive compounds they contain [51]. For instance, a study by Kakudidi et al. [19, 52] in southwestern Uganda showed that plants like *Tetradenia riparia* had high antifungal activity against *C. albicans*. This was attributed to the various bioactive compounds like steroidal glycosides, coumarins and tannins it contained. Also, a study by Kamatenesi-Mugisha et al. [53] in and around Queen Elizabeth Biosphere Reserve, in western Uganda reported *Allium sativum* to be fungicidal to oral candidiasis. *Solenostemon latifolius* (Hochst. Ex. Benth) JK Morton, *Hoslundia opposita* Vahl, *Azadirachta indica* A Juss. and *Rumex usambarensis* Dammer are documented as potential anti-candida plants in Njeru sub-county, Buikwe district, central Uganda [54]. Additionally, a study conducted in Uganda by Anywar et al. [16] documented *Chamaecrista absus* (L.) H.S. Irwin & Barneby as a potential anti-candida plant.

Besides, these ethnomedicinal plants are cheap and easily available [55]. Therefore, plants documented in this study could potentially reduce the candidiasis burden in Pader district since the community can readily access them.

The herbalists had equal chances of treating candidiasis patients regardless of their gender, age, education level, occupation, marital status and religion. However, the information on herbal medicine was still secretive, mostly inherited or shared among herbalists. This was also cited by Ozioma and Chinwe [56] who stated that herbalists pass information to few trusted relatives and associates; thus, documentation will increase information access and usage by the community. Intellectual knowledge of herbalists must be acknowledged [57].

### Medicinal plants as treatment options for candidiasis

The herbalists’ preference of herbal to western medication for treatment of candidiasis is corroborated by the results of FGDs (non-herbalists). *“With herbal medicine, the child recovers in just one week, so everyone who wishes quick recovery of their children goes for herbal treatment option”* (FGD, Ogom HCII). This could be due to limited access to health care services. Around 31.6% population of Pader district can hardly access health services [31]. A report from MOH [58] indicated that the biggest community Health Centre in Pader district is Pajule Health Centre (HC) IV. The others are at the level of HCs III and II.

Findings showed that females preferred herbal to western medication possibly because genitourinary candidiasis is more prevalent in females than men. About 50% of women, mostly in the reproductive age bracket get multiple infection episodes of VVC [59] and thus, the great demand for herbal remedies. Vaginal infections like VVC affect female private parts [60]. Culturally, this makes it difficult for the females to consult especially male medics, thus they opt for herbal treatment. Furthermore, field observations showed that females are more responsible for nursing the sick members of their families, especially those with compromised immunity like HIV/AIDS patients. They frequently suffer from opportunistic diseases like OPC [61]. Kachmar et al. [62] attributed females’ preference of herbal to western medication to their nature of work of being in charge of obtaining and preparation of medicinal plants remedies, for the health care of the family members. Additionally, Purba and Febriani [63] noted that women have larger social networks that are used to get information. For instance, Welz et al. [64], showed that dissatisfaction with conventional treatment was the most important reason for use of herbal remedies. There are many reported cases of conventional drug failures due to drug resistance [12, 65]. Of late, a lot of scientific investigations have been carried out on herbal medicine confirming their efficacy in certain conditions hence the several phyto-pharmaceutical products on the market [56].

#### Harvesting time of medicinal plants

Harvesting time has an impact on drug potency. A study by Black et al. [66] showed that harvesting time of *R. tomentosum* impacted on its phenolic content and bioactivity. Although some scholars have stated that harvesting time of plants for medicinal purpose is species specific [67, 68], others like Papadopoulos et al. [69] suggested that, generally it should be done early mornings immediately the dew on the plants dries off, when the concentration of the bioactive molecules in plants are at their peak. This is because high moisture contents of the plants can stimulate microbial fermentation and growth of molds that may make the herbs become harmful [70, 71]. The choice of harvesting in the morning by some of the herbalists can thus be supported by scientific evidence.

#### Source of knowledge for potential anti-candida herbal medicine

Knowledge on potential anti-candida herbal medicine in Pader district is orally passed from generation to generation, thus such knowledge is at the risk of being lost due to lack of documentation [72]. Transmission of indigenous knowledge in Pader district on potential anti-candida plants could be attributed to disease burden in the area. Besides, there is high dependency of these rural populations on herbal medicine for primary health care needs due to inaccessible health care services. Many homes are located 5 km and beyond from the nearest health centers [31]. Although there is a good practice of knowledge transfer by herbalists, the information still remains oral and secretive. It is done within families and among fellow herbalists, and not open to the general public, including research institutions [73].

### Medicinal plants usage for management of common fungal infections

#### Informant consensus factor (FIC)

There was high consensus among herbalists on herbal remedies for both OPC and VVC (FIC = 0.9) (Table 5). Furthermore, there was consensus from the FGD held in Pajule HCIV that all these herbal remedies are good. This shows that there is agreement among the herbalists and the community on the medicinal plants for the management and treatment of candidiasis. OPC and VVC are caused by *Candida* spp [74, 75], thus similar plants are used in their management and treatment. Chekole [76] pointed out that values of FIC indicate the magnitude of shared knowledge of the most important herbal plant species for treating diseases that are prevalent in the community. Similarly, Mengistu et al. [43], stated that high values of FIC above 0.7 indicate high levels of agreement of traditional knowledge usage of medicinal plants.

#### Medicinal plants for treatment of candidiasis and their effectiveness

Although some of the widely used potential anti-candida plants in this study have not been reported in literature for their anti-candida potentials, different studies revealed that they contain several specific bioactive compounds that makes them potential therapeutic plants. The three most frequently used potential anti-candida plant species in Pader district were *Momordica foetida*, *Sansevieria dawei* and *Khaya anthotheca*. A systematic review by Muronga et al. [77] indicated that *Momordica foetida* contains various curative compounds like phenolic glycosides and alkaloids which exhibited diverse medicinal values against various diseases. For instance, Kamatenesi‐Mugisha et al. [53] documented *Momordica foetida* as one of the potential anti-fungal plants in and around Queen Elizabeth Biosphere Reserve in western Uganda*.* Namukobe et al. [78] and Omara et al. [79] documented *Sansevieria dawei* as a plant with medicinal purposes due to various phytocompunds like saponins, terpenoids and flavonoids. *Sansevieria hyacinthoides* demonstrated antifungal activity against *Candida albicans* [80, 81]. It was also reported that *Khaya anthotheca* contains various limonoids that demonstrate an extensive range of biological activities that promote health in living organisms. Hamza et al. [82] reported antifungal activity of methanolic *Khaya anthotheca* extract against *Candida krusei*. *Khaya ivorensis* exhibited significant antifungal activity against the plant pathogenic fungus (*Botrytis cinerea* Pers.) [83]. Also, *Solanecio mannii* in this study exhibited anti-candida activity [52] from southwestern Uganda. Thus, for some of the documented plant species whose anti-candida activities are not yet reported, bioactivity studies based on their FIC should be carried out for their validation as potential anti-candida therapies.

In this study, roots and barks of the plants were commonly used possibly because they are sites with more bioactive compounds. Szwajkowska-Michałek et al. [84] stated that most of the secondary metabolites containing the bioactive compounds are located in vacuoles and cell walls of peripheral tissues. Wei et al. [85] discovered various phytochemicals from root barks of Morus species, with variety of antimicrobial potentials. Similarly, Lezoul et al. [86] compared the total bioactive compounds in organs of three medicinal plants, viz *Passiflora caerulea*, *Physalis peruviana* and *Solanum muricatum*. Their leaves and roots contained higher concentrations of bioactive compounds than other organs.

#### Sustainable utilization of medicinal plant species

Uprooting whole plants and cutting the plant parts of interest, like the main roots (Table 6), are inappropriate harvesting methods that threatens the existence of these plants [87], thus, need for sustainable utilization of these plants [88]. Khumalo et al. [67] advised herbalists to use plant parts, for instance lateral roots, which must be dug out at distance of about 30 cm from the main root/ stem. Ring barking must be avoided to avoid death of the plants. Instead, small sections of the barks could be removed. Plants obtained from the wild can easily be lost due to lack of monitoring and maintenance [89]. This therefore calls for conservation measures for seasonal medicinal plants to increase on their availability for use by the community.

#### Modes of preparation and administration of potential anti-candida plants

Decoction and maceration were commonly used (Table 6) because heat produced during decoction increases the solubility of herbal compounds, and hence their easy transfer from plant materials to the solvent. Decoctions are suited for extraction of thermal stable compounds. Maceration is one of the simplest and widely used methods. It operates on the principle of diffusion. However, it requires a lot of time to allow the molecules to diffuse from the plant materials to the solvent [90]. The use of polar solvents such as water is common practice in extraction of compounds from medicinal plants [91]. S*ansevieria dawei, Momordica foetida* and *Hallea rubrostipulata* were mixed together to increase their synergy [92]. The herbalists are knowledgeable on herbal medicinal properties, including steps that can extract the curative compounds [56]. This gives high degree of authenticity to the research findings of potential anti-candida plants.

Oral and anal routes of herbal administration being preferred to orthodox medicine could be related to the nature of bioactive compounds in those plants. For instance, alkaloids are easily absorbed when orally taken [93]. Different portions of the small intestine (duodenum, jejunum and ileum) play particular roles in drug assimilation and with special absorptive capabilities [94]. However, the biggest challenge with medicinal plants usage is lack of standardization that jeopardizes quality control and safety. Furthermore, the disposable syringes may be shared and are not sterilized, hence high chances of disease transmission. The integration of herbal medicine into the health care system, will promote use of safe, cheap and easily accessible health care service provision. Thus, this trend will result in increased economic potential and poverty reduction among the communities [56].

### Limitations of the study

The major limitation of this study was failure to collect fertile plant specimens of a few plants species that were not in flowering/fruiting stage. These plants could not be identified and so local names were used.

## Conclusion

The community of Pader district has rich indigenous knowledge on candidiasis which is troublesome in the area, and they prefer herbal remedies to manage the infections compared to orthodox treatment**.** This has made people to transfer the knowledge from generation to generation, though the practice is still secretive among relatives and herbalists. The herbalists mostly use unsustainable harvesting techniques like uprooting whole plants and cutting of roots. Therefore, there is need for community sensitization on candidiasis herbal remedies and sustainable harvesting of the plants. This study recommends that the use of herbal medicine as alternative treatment option for candidiasis should be supported by the government of Uganda through standardizing herbal remedies to improve on their quality; this will provide cheaper health care option and also widen the knowledge base among the locals to meet their primary health care needs. This is in line with Sustainable Development Goal, agenda 3, of ensuring healthy lives and promoting well-being for all by 2030. The study further recommends putting in place practical conservation measures to conserve medicinal plants in Pader district. Further studies should be conducted on the mentioned plant species to verify their anti-candida potentials and safety.

## Data Availability

Data and materials of this study will be available upon request.

## Abbreviations

MFPED: Ministry of Finance, Planning and Economic Development
OPC: Oropharyngeal candidiasis
VVC: Vulvovaginal candidiasis
FIC: Informant Consensus Factor
FGD: Focus group discussions
ODK: Open data kit
UBOS: Uganda Bureau of Statistics
MOH: Ministry of Health

## References

[1] Bongomin F, Gago S, Oladele RO, Denning DW (2017). Global and multi-national prevalence of fungal diseases—estimate precision. J fungi.

[2] Brown GD, Denning DW, Gow NAR, Levitz SM, Netea MG, White TC (2012). Hidden killers: human fungal infections. Sci. Transl. Med..

[3] Kmeid J, Jabbour J-F, Kanj SS (2020). Epidemiology and burden of invasive fungal infections in the countries of the Arab League. J Infect Public Health.

[4] Ravikumar S, Win MS, Chai LYA (2015). Optimizing outcomes in immunocompromised hosts: understanding the role of immunotherapy in invasive fungal diseases. Front Microbiol.

[5] Govender NP, Chiller TM, Poonsamy B, Frean JA (2011). Neglected fungal diseases in sub-Saharan Africa: a call to action. Curr Fungal Infect Rep.

[6] Bamba S, Zida A, Sangaré I, Cissé M, Denning DW, Hennequin C (2018). Burden of severe fungal infections in Burkina Faso. J Fungi.

[7] Parkes-Ratanshi R, Achan B, Kwizera R, Kambugu A, Meya D, Denning DW (2015). Cryptococcal disease and the burden of other fungal diseases in Uganda; where are the knowledge gaps and how can we fill them?. Mycoses.

[8] Rubaihayo J, Tumwesigye NM, Konde-Lule J, Wamani H, Nakku-Joloba E, Makumbi F (2016). Frequency and distribution patterns of opportunistic infections associated with HIV/AIDS in Uganda. BMC Res Notes.

[9] Achkar JM, Fries BC (2010). Candida infections of the genitourinary tract. Clin Microbiol Rev.

[10] Sobel JD (2007). The emergence of non-albicans Candida species as causes of invasive candidiasis and candidemia. Curr Fungal Infect Rep.

[11] Kowalsky SF, Dixon DM (1991). Fluconazole: a new antifungal agent. Clin Pharm.

[12] Varadarajan S, Narasimhan M, Malaisamy M, Duraipandian C (2015). In vitro anti-mycotic activity of hydro alcoholic extracts of some Indian medicinal plants against fluconazole resistant *Candida albicans*. J Clin Diagn Res JCDR.

[13] Kołaczkowska A, Kołaczkowski M (2016). Drug resistance mechanisms and their regulation in non-albicans Candida species. J Antimicrob Chemother.

[14] WHO. Traditional medicine strategy 2002 2005. World Health Organisation, Geneva, 2002.

[15] Schultz F, Anywar G, Wack B, Quave CL, Garbe L-A (2020). Ethnobotanical study of selected medicinal plants traditionally used in the rural Greater Mpigi region of Uganda. J Ethnopharmacol.

[16] Anywar G, Kakudidi E, Byamukama R, Mukonzo J, Schubert A, Oryem-Origa H (2020). Indigenous traditional knowledge of medicinal plants used by herbalists in treating opportunistic infections among people living with HIV/AIDS in Uganda. J Ethnopharmacol.

[17] Asiimwe S, Kamatenesi-Mugisha M, Namutebi A, Borg-Karlsson A-K, Musiimenta P (2013). Ethnobotanical study of nutri-medicinal plants used for the management of HIV/AIDS opportunistic ailments among the local communities of western Uganda. J Ethnopharmacol.

[18] Mugisha MK, Asiimwe S, Namutebi A, Borg-Karlson A-K, Kakudidi EK (2014). Ethnobotanical study of indigenous knowledge on medicinal and nutritious plants used to manage opportunistic infections associated with HIV/AIDS in western Uganda. J Ethnopharmacol.

[19] Kakudidi E, Anywar G, Fredrick A, Jasper O-O (2015). Antifungal medicinal plants used by communities adjacent to Bwindi impenetrable National Park, South-Western Uganda. Eur J Med Plants.

[20] Tugume P, Nambejja C, Nyakoojo C, Kamatenesi-Mugisha M (2019). Medicinal plant species used in the treatment of skin diseases in Katabi Subcounty, Wakiso District, Uganda. Ethnobot Res Appl.

[21] Guto JA, Bii CC, Denning DW (2016). Estimated burden of fungal infections in Kenya. J Infect Dev Ctries.

[22] Nannyonjo J (2005). Conflicts, poverty and human development in Northern Uganda. Round Table.

[23] Nobile CJ, Johnson AD (2015). *Candida albicans* biofilms and human disease. Annu Rev Microbiol.

[24] Namakula J, Witter S (2014). Living through conflict and post-conflict: experiences of health workers in northern Uganda and lessons for people-centred health systems. Health Policy Plan.

[25] Marahatta SB (2020). Barriers in the access, diagnosis and treatment completion for tuberculosis patients in central and western Nepal: a qualitative study among patients, community members and health care workers. PLoS ONE.

[26] Ekor M (2014). The growing use of herbal medicines: issues relating to adverse reactions and challenges in monitoring safety. Front Pharmacol.

[27] Kyoshabire M, Katuura E, Cunningham AB, Hoeft R (2017). Medicinal plants and herbalist preferences around Bwindi Impenetrable National Park. J Med Plants Res.

[28] Katuura E, Waako P, Ogwal-Okeng J, Bukenya-Ziraba R (2007). Traditional treatment of malaria in Mbarara District, western Uganda. Afr J Ecol.

[29] Cheikhyoussef A, Mapaure I, Shapi MK. The use of some indigenous plants for medicinal and other purposes by local communities in Namibia with emphasis on Oshikoto region: a review. 2011.

[30] MFPED. Uganda Poverty Status Report 2014. pp. 1–96, 2014.

[31] UBOS. The national population and housing census 2014, area specific profile series, Kampala, Uganda. Uganda Bureau of Statistics Kampala, Uganda, Uganda Bureau of Statistics. (2017). The national population and housing Kampala, Uganda. 2017.

[32] Anywar G, Kakudidi E, Byamukama R, Mukonzo J, Schubert A, Oryem-Origa H (2020). Medicinal plants used by traditional medicine practitioners to boost the immune system in people living with HIV/AIDS in Uganda. Eur J Integr Med.

[33] Nyumba TO, Wilson K, Derrick CJ, Mukherjee N (2018). The use of focus group discussion methodology: Insights from two decades of application in conservation. Methods Ecol Evol.

[34] Martin GJ (1995). Ethnobotany: a methods manual.

[35] Tade O (2013). A spiritual dimension to cybercrime in Nigeria: the ‘yahoo plus’ phenomenon. Hum Aff.

[36] Masoga MA, Shokane AL (2020). Socio-economic challenges faced by traditional healers in Limpopo province of South Africa: conversations from below. Altern An Int J Indig Peoples.

[37] Jaradat NA, Ayesh OI, Anderson C (2016). Ethnopharmacological survey about medicinal plants utilized by herbalists and traditional practitioner healers for treatments of diarrhea in the West Bank/Palestine. J Ethnopharmacol.

[38] Bowen GA (2008). Naturalistic inquiry and the saturation concept: a research note. Qual Res.

[39] Human Services Research Institute. North Dakota behavioral health system study | Final Report. no. April, pp. 200–250, 2018.

[40] Stalmeijer RE, McNaughton N, Van Mook WNKA (2014). Using focus groups in medical education research: AMEE Guide No. 91. Med Teach.

[41] Randrianarivony TN (2017). The most used medicinal plants by communities in Mahaboboka, Amboronabo, Mikoboka, Southwestern Madagascar. J Ethnobiol Ethnomed.

[42] Trotter RT, Logan MH, Etkin NL (1986). Informant consensus: a new approach for identifying potentially effective medicinal plants. Plants in indigenous medicine & diet.

[43] Mengistu DK, Mohammed JN, Kidane YG, Fadda C (2022). Diversity and traditional use knowledge of medicinal plants among communities in the south and south-eastern zones of the Tigray Region, Ethiopia. Diversity.

[44] Omara P, Akwongo B (2022). Learning to teach in the era of uncertainties: challenges and lessons learnt by student teachers during COVID-19 pandemic in Uganda. Eur J Educ Stud.

[45] Burnard P, Gill P, Stewart K, Treasure E, Chadwick B (2008). Analysing and presenting qualitative data. Br Dent J.

[46] Nyirjesy P, Peyton C, Weitz MV, Mathew L, Culhane JF (2006). Causes of chronic vaginitis: analysis of a prospective database of affected women. Obstet Gynecol.

[47] Benzie IFF, Wachtel-Galor S. Herbal medicine: biomolecular and clinical aspects. 2011. 22593937

[48] W. MOH Uganda, Health and mortality survey among internally displaced persons in Gulu, Kitgum and Pader districts, Northern Uganda. World Health Organization, 2005.

[49] Montoya MC, Moye-Rowley WS, Krysan DJ (2019). Candida auris: the canary in the mine of antifungal drug resistance. ACS Infect Dis.

[50] Omona S, Malinga GM, Opoke R, Openy G, Opiro R (2020). Prevalence of diarrhoea and associated risk factors among children under five years old in Pader District, northern Uganda. BMC Infect Dis.

[51] Roy A (2021). Hairy root culture an alternative for bioactive compound production from medicinal plants. Curr Pharm Biotechnol.

[52] Kakudidi E, Ayorekire F, Ogwal-Okeng J, Anywar G. Phytochemical analysis and screening of ugandan medicinal plants for antifungal activity against *Candida albicans*. 2015.

[53] Kamatenesi-Mugisha M, Oryem-Origa H, Odyek O, Makawiti DW (2008). Medicinal plants used in the treatment of fungal and bacterial infections in and around Queen Elizabeth Biosphere Reserve, western Uganda. Afr J Ecol.

[54] Shehu MW, Bello I, Abdulkadir N, Shehu A, Jamil SE, Waziri SA (2018). Utilization of medicinal plants used in the management of HIV/AIDS opportunistic infections in Njeru sub-county, Buikwe district, Uganda. MOJ Bioequiv Availab.

[55] Roy M (2022). Traditional homegardens and ethnomedicinal plants: Insights from the Indian Sub-Himalayan region. Trees For People.

[56] Ozioma E-OJ, Chinwe OAN (2019). Herbal medicines in African traditional medicine. Herb Med.

[57] Oliva MJ, Rukundo O. A guide to intellectual property issues in access and benefit-sharing agreements, vol. 1052. WIPO, 2018.

[58] MOH. National Health Facility Master List 2018, A complete list of All Health Facilities in Uganda. Ministry of Health Kampala, Uganda, 2018.

[59] Mbakwem-Aniebo C, Osadebe AU, Athanasonny E, Okonko IO (2020). Prevalence of Candida spp. and age-related disparities amongst women presenting with vaginitis at the Obstetrics and Gynaecology (O&G) Clinic in a Tertiary hospital in Port Harcourt, Nigeria. Afr Health Sci.

[60] Adolfsson A, Hagander A, Mahjoubipour F, Larsson P-G (2017). How vaginal infections impact women’s everyday life: women’s lived experiences of bacterial Vaginosis and recurrent vulvovaginal candidiasis. Adv Sex Med.

[61] Ambe NF et al. The prevalence, risk factors and antifungal sensitivity pattern of oral candidiasis in HIV/AIDS patients in Kumba District Hospital, South West Region, Cameroon. Pan Afr Med J. 2020;36(1). 10.11604/pamj.2020.36.23.18202 PMC7392032 32774600

[62] Kachmar MR, et al. Traditional knowledge of medicinal plants used in the Northeastern part of Morocco. Evidence-Based Complement Altern Med. 2021; 2021. 10.1155/2021/6002949 PMC8426073 34512779

[63] Purba MM, Febriani I. Family support in caring for people with mental disorders at the menteng health center palangka raya city. in International Conference on Nursing and Public Health, 2021; 1(1): 115–119.

[64] Welz AN, Emberger-Klein A, Menrad K (2018). Why people use herbal medicine: insights from a focus-group study in Germany. BMC Complement Altern Med.

[65] Ma Q, Wei Y, Meng Z, Chen Y, Zhao G (2022). Effects of water extract from Artemisia argyi leaves on LPS-induced mastitis in mice. Animals.

[66] Black P (2011). Seasonal variation of phenolic constituents and medicinal activities of Northern Labrador tea, Rhododendron tomentosum ssp. subarcticum, an Inuit and Cree First Nations traditional medicine. Planta Med.

[67] Khumalo S, Fröde A, Sola P. Guidelines for the sustainable harvesting of traditional medicinal plants in Zimbabwe. Harare Zimbabwe Minist Environ Tour. 2019.

[68] Figueiredo AC, Barroso JG, Pedro LG, Scheffer JJC (2008). Factors affecting secondary metabolite production in plants: volatile components and essential oils. Flavour Fragr J.

[69] Papadopoulos AP, et al. Soilless greenhouse production of medicinal plants in North Eastern Canada. In World Congress on Soilless Culture: Agriculture in the Coming Millennium 554, 2000, pp. 297–304.

[70] Pandey AK, Savita R (2017). Harvesting and post-harvest processing of medicinal plants: problems and prospects. Pharma Innov J.

[71] Organización Mundial de la Salud and WHO, *WHO guidelines on good agricultural and collection practices [GACP] for medicinal plants*. World Health Organization, 2003.

[72] Gakuya DW (2020). Traditional medicine in Kenya: past and current status, challenges, and the way forward. Sci African.

[73] Mintah SO (2022). Medicinal plant use in Ghana: advancement and challenges. Am J Plant Sci.

[74] Song N (2022). A prospective study on vulvovaginal candidiasis: multicentre molecular epidemiology of pathogenic yeasts in China. J Eur Acad Dermatology Venereol.

[75] Solis NV (2022). Systematic genetic interaction analysis identifies a transcription factor circuit required for oropharyngeal candidiasis. MBio.

[76] Chekole G (2017). Ethnobotanical study of medicinal plants used against human ailments in Gubalafto District, Northern Ethiopia. J Ethnobiol Ethnomed.

[77] Muronga M (2021). Three selected edible crops of the genus Momordica as potential sources of phytochemicals: biochemical, nutritional, and medicinal values. Front Pharmacol.

[78] Namukobe J, Lutaaya A, Asiimwe S, Byamukama R. An ethnobotanical study of medicinal plants used in the management of dermatological disorders in Buyende and Kayunga Districts, Uganda. 2021.

[79] Omara T (2020). Antivenin plants used for treatment of snakebites in Uganda: ethnobotanical reports and pharmacological evidences. Trop Med Health.

[80] Maroyi A (2019). Sansevieria hyacinthoides (L.) Druce: a review of its botany, medicinal uses, phytochemistry, and biological activities. Asian J Pharm Clin Res.

[81] Sultana N (2011). Antimicrobial compounds from the Rihzomes of Sansevieria hyacinthoides. Bangladesh J Sci Ind Res.

[82] Hamza OJM (2006). Antifungal activity of some Tanzanian plants used traditionally for the treatment of fungal infections. J Ethnopharmacol.

[83] Olatunji TL, Odebunmi CA, Adetunji AE (2021). Biological activities of limonoids in the Genus Khaya (Meliaceae): a review. Futur J Pharm Sci.

[84] Szwajkowska-Michałek L, Przybylska-Balcerek A, Rogoziński T, Stuper-Szablewska K (2020). Phenolic compounds in trees and shrubs of central Europe. Appl Sci.

[85] Wei H, Zhu J-J, Liu X-Q, Feng W-H, Wang Z-M, Yan L-H (2016). Review of bioactive compounds from root barks of Morus plants (Sang-Bai-Pi) and their pharmacological effects. Cogent Chem.

[86] Lezoul NEH, Belkadi M, Habibi F, Guillén F (2020). Extraction processes with several solvents on total bioactive compounds in different organs of three medicinal plants. Molecules.

[87] Kimondo J, Miaron J, Mutai P, Njogu P (2015). Ethnobotanical survey of food and medicinal plants of the Ilkisonko Maasai community in Kenya. J Ethnopharmacol.

[88] Soetan KO, Aiyelaagbe OO (2009). The need for bioactivity-safety evaluation and conservation of medicinal plants-a review. J Med plants Res.

[89] Güler B, Manav E, Uğurlu E (2015). Medicinal plants used by traditional healers in Bozüyük (Bilecik–Turkey). J Ethnopharmacol.

[90] Stéphane FF, Jules BK, Batiha GE, Ali I, Bruno LN. Extraction of bioactive compounds from medicinal plants and herbs. Nat Med Plants. 2021.

[91] Abubakar AR, Haque M (2020). Preparation of medicinal plants: Basic extraction and fractionation procedures for experimental purposes. J Pharm Bioallied Sci.

[92] Archana H, Bose VG (2022). Evaluation of phytoconstituents from selected medicinal plants and its synergistic antimicrobial activity. Chemosphere.

[93] Boadu AA, Asase A. Documentation of herbal medicines used for the treatment and management of human diseases by some communities in southern Ghana. Evidence-Based Complement Altern Med. 2017; 2017. 10.1155/2017/3043061 PMC5480049 28684965

[94] Liu J-Y (2013). Intestinal absorption and bioavailability of traditional Chinese medicines: a review of recent experimental progress and implication for quality control. J Pharm Pharmacol.

[95] Ullrich H. Traditional knowledge, biodiversity, benefit-sharing and the patent system: romantics v. economics? Economics. 2005.

